# Chemical Analysis and Antioxidant and Antimicrobial Activity of Essential oils from *Artemisia negrei* L. against Drug-Resistant Microbes

**DOI:** 10.1155/2021/5902851

**Published:** 2021-09-07

**Authors:** Khalid Chebbac, Abdelfattah EL Moussaoui, Mohammed Bourhia, Ahmad Mohammad Salamatullah, Abdulhakeem Alzahrani, Raja Guemmouh

**Affiliations:** ^1^Laboratory of Biotechnology Conservation and Valorisation of Natural Resources, Faculty of Sciences Dhar ElMahraz, Sidi Mohammed Ben Abdallah University, Fez, Morocco; ^2^Laboratory of Biotechnology, Environment, Agri-food, and Health, University of Sidi Mohamed Ben Abdellah, Faculty of Sciences Dhar ElMahraz, Fez, Morocco; ^3^Laboratory of Chemistry-Biochemistry, Environment, Nutrition, and Health, Faculty of Medicine and Pharmacy, Hassan II University, Casablanca, B.P 5696, Casablanca, Morocco; ^4^Department of Food Science & Nutrition, College of Food and Agricultural Sciences, King Saud University, P.O. Box 2460, Riyadh 11451, Saudi Arabia

## Abstract

**Background:**

*Artemisia negrei* L. (*A. negrei*) is a medicinal and aromatic plant belonging to the family Asteraceae that is more widespread in the folded Middle Atlas Mountains, Morocco.

**Materials and Methods:**

This study was run to investigate the phytochemical composition and antioxidant, antibacterial, and antifungal activities of *Artemisia negrei* L. essential oil. This oil was extracted from the fresh plant material by using the Clevenger apparatus. The phytochemical composition was characterized by GC-MS. The antioxidant activity was evaluated using different methods including DPPH, *β*-carotene bleaching, and total antioxidant capacity. The antibacterial activity was tested vs. multidrug-resistant bacteria including both Gram-negative and Gram-positive using inhibition zones in agar media and minimum inhibitory concentration (MIC) bioassays. The antifungal activity was conducted on *Candida albicans*, *Aspergillus niger*, *Aspergillus flavus,* and *Fusarium oxysporum* using a solid medium assay.

**Results:**

The chromatographic characterization of essential oils of *A. negrei* revealed the presence of 34 compounds constituting 99.91% of the total essential oil. The latter was found to have promising antioxidant activity by all bioassays used such as DPPH, *β-*carotene bleaching, and total antioxidant capacity. The results obtained showed that our plant oils had potent antibacterial activity towards Gram-negative (*E. coli* 57, *E. coli* 97, *K. pneumonia*, and *P. aeruginosa)* and Gram-positive (*S. aureus*), so that the maximum inhibition zones and MIC values were around 18–37 mm and 3.25 to 12.5 mg/mL, respectively. The oil also showed antifungal activity towards *Candida albicans*, *Fusarium oxysporum,* and *Aspergillus Niger* except for flavus species.

**Conclusion:**

The findings obtained in the work showed that *A. negrei* can serve as a valuable source of natural compounds that can be used as a new weapon to fight radical damage and resistant microbes.

## 1. Introduction

Herbal medicine has become a more popular way of fighting against diseases and producing pharmaceutical medicines [1–3]. The use of herbal medicine for seeking potentially active compounds has been proven to promote scientific output [4]. Many synthesized drugs have come from natural sources including medicinal plants which can be available in the form of food supplements, nutraceuticals, and alternative and complementary medicines [5]. Plants are an important source of natural substances with great antioxidant potential [6]. Modern medicines place in priority the development of effective antioxidant substances from a natural source for being applied in the medical field for medication purposes [7]. Natural antioxidant agents received full consideration in the food industry to prevent oxidative deterioration of food by free radicals. These agents have been placed in priority for being used as an alternative to synthetic antioxidant agents such as butylated hydroxyanisole (BHA), butylated hydroxytoluene (BHT), and tertbutylhydroquinone (TBHQ) that are suspected of having serious side effects including carcinogenic and toxic effects [8].

Species among the genus *Artemisia* are used in traditional medicines for therapeutic purposes as antispasmodic, antirheumatic, antiinflammatory, antimicrobial, antihelminthics, and antiveinous agents [9, 10]. *Artemisia negrei* L. is a medicinal plant endemic to Morocco lands, which is distributed in the region of the Moulouya Basin, and the folded Middle Atlas Mountains of Morocco. This plant is commonly used in Moroccan traditional medicines in treating diseases including digestive genital tracts and dermatological infections by using powder and infusion forms [9–11].

Antimicrobial resistance is a phenomenon where microbes evolve strategies to fight against drugs planned to attack them, so that the germs, which are not defeated, continue to develop powerfully as never before [7]. A few years ago, antimicrobial resistance has become one of the biggest problems that overburden the health care system and is classified as among the greatest challenge by the World Health Organization for 2019 [12].

In this study, the studied bacterial strains belong to drug-resistant microbes such as *P. aeruginosa, K. pneumonia, S. aureus,* and *E. coli* pathogens. It is well known that these species can be multidrug-resistant [13]. Some of the fungal species studied in this work belong to drug-resistant microbes such as *Candia* spp., which was involved in the infection of more than 90% of people with AIDS in an earlier time. *Candia* spp. has developed unprecedented resistance due to excessive use of synthesized drugs to fight fungal infections caused by these microbes and continues to be a greatest growing health burden [14].

The current research study aimed to investigate the phytochemical composition and antioxidant, antifungal, antibacterial, and antifungal activities of *A. negrei* essential oil since no other studies have attempted this objective up to the time of writing this article.

## 2. Materials and Methods

### 2.1. Selection and Identification of Plant Material

*A. negrei* was harvested in June 2019 from the Middle Folded Atlas, Morocco (2100 m, 33.539648, −3.894474). The authentification was done by a botanist with reference # BPRN/04/18 that was deposited at the herbarium of Sidi Mohammed Ben Abdallah University, Fez, Morocco. Next, aerial parts of *A. negrei* were subjected to dry in a ventilated place for 10 days before extraction.

### 2.2. Extraction of Essential Oil

In the present work, the oil was extracted from the fresh plant material by using the Clevenger apparatus. In brief, a total of 200 g of aerial parts (leaves) were cut into small pieces before being placed into a flask with 750 mL of distilled water. Afterward, the whole solution was boiled for 3 h to maximize the essential oil extraction. The essential oil yield was obtained by using the following formula:

(1) $$\begin{array}{c} \text{RHE}=\frac{M{}^{\prime }}{M}\times 100, \end{array}$$

where RHE is essential oil yield in %; *M*' is essential oil mass recovered in grams; *M* is plant material mass in grams.

#### 2.2.1. Analysis of the Phytochemical Composition of the Oil

The phytochemical characterization of essential oil was effectuated by GC-MS using a nonpolar silica column. To fulfill this goal, the operating conditions of the analysis were run as follows: the initial temperature was set to 40°C/2 min along with speed 2°C/min, while the final and injector temperatures were set to 260°C/10 min and 250°C, respectively. In this analysis, helium gas was used as a vehicle (1 mL/min) with “split” mode injection. The ionization energy and ion source temperature were 70 eV and 200°C, respectively, and the scan mass range m/z is 40–650. The oil was diluted in hexane solvent (10 : 100) before being injected with 1 *µ*L. The chemical identification was done by using retention indices (RI) along with comparison with ADAMS database [15].

### 2.3. Antioxidant Activity

In this study, the antioxidant power of the oil from *A. negrei* was evaluated using three bioassays including DPPH, *β*-carotene bleaching, and total antioxidant capacity [16].

#### 2.3.1. DPPH Radical Scavenging Activity

DPPH bioassay was carried out using protocols as reported by Tepe et al. [17]. Both the essential oil (EO) and the positive control (BHT) were used at different concentrations including 1, 1/4, 1/8, 1/16, 1/32, 1/64, 1/128, 1/256, and 1/512 mg/mL. The anti-free radical activity was evaluated by mixing 100 *µ*L of each previously prepared concentration (EO and BHT) with 750 *µ*L of DPPH (0.004%). Afterward, the solution was incubated at ambient temperature for 30 minutes before reading the absorbance. The DPPH scavenging ability was expressed as inhibition percentage as follows:

(2) $$\begin{array}{c} \mathrm{PI}\left(\%\right)=\left(A0-A/A0\right)*100, \end{array}$$

where PI is the percentage of inhibition, *A*0 is the absorbance of DPPH without the sample (control), and *A* the absorbance of DPPH with the sample.

#### 2.3.2. Total Antioxidant Capacity Test (TAC)

The TAC test was carried out according to the protocol reported in the earlier work [18]. In brief, 25 *µ*L of the sample test (1 mg/mL) was mixed with one milliliter of reagent solution constituted of sodium phosphate, sulphuric acid, and ammonium molybdate. Next, the whole solution was placed for incubation at 95°C for 90 min before measuring the absorbance at 695 nm using a spectrophotometer [18]. BHT and Quercetin were used as standard references. The TAC has expressed in mg EAA/g HE.

### 2.4. *ß*-Carotene Bleaching Assay

This assay was performed to study the antioxidant power of essential oil from *A. negrei* using the protocol as reported in the literature [19, 20]. In brief, 1 mL of *ß*-carotene chloroform solution was added to 10 *µ*L of solution constituted of linoleic acid and 100 mg of Tween 80. Next, the chloroform was retrieved using a vacuum rotary evaporator before adding 25 mL of hydrogen peroxide to the residue. Afterward, 2.5 mL of the obtained mixture was added to 100 *µ*L of the sample test (1 mg/mL) and then maintained in the water bath at 51°C for 2h. BHT was used as a standard reference (1 mg/mL). The absorbance was measured at 470 nm. The antioxidant power was calculated as a percentage of antioxidant activity relative to the control as follows:

(3) $$\begin{array}{c} \mathrm{AA}\%=\left(\mathrm{AE}/\mathrm{ABHT}\right)*100. \end{array}$$

AA% is the antioxidant property percentage, and ABHT is the absorbance of the positive control, while AE is the absorbance of the negative control.

### 2.5. Antibacterial Activity

The evaluation of the antimicrobial activity of the essential oils was carried out according to the previously reported data elsewhere [16]. The essential oil of *A. negrei* was tested vs. Gram-negative bacteria *Pseudomonas aeruginosa, Escherichia coli* ATB:57; *Klebsiella pneumoniae*, and *Escherichia coli* ATB:97 and Gram-positive bacteria (*Staphylococcus aureus* (*LM*, *FMP*, and *Fez*)). The strains tested in the current study were clinically isolated and have been reported as multidrug-resistant as reported in earlier work [13, 21, 22]. The bacterial suspension was prepared from fresh culture. To achieve this goal, few colonies from the culture were aseptically seeded in 0.9% of physiological water at a density of 0.5 McFarland, which corresponded to 10^7^ to 10^8^ CFU/mL [23].

The antibacterial activity was studied using the disc diffusion method. In brief, a volume of 10 *µ*L of *A. negrei* essential oil (1 mg/mL) was used for testing purposes, while ampicillin 1.68 mg/disc and streptomycin 0.020 mg/disc were used as drug references as reported in earlier work [20].

The minimum inhibitory concentrations (MICs) were studied by using the microdilution assay [24]. In brief, MIC was assessed by using the microdilution method in 96-well plates. The concentrations of the oil were prepared in a 0.2% agar suspension. The concentrations were obtained by successive dilutions (25 to 0.02 mg/mL). Finally, the plates were placed for incubation at 37 C for 18 h. Next, the bacterial growth was visualized after adding 20 *µ*L of triphenyltetrazolium in 5 mg/mL wells before further incubation for 30 min at 37°C [23].

### 2.6. Antifungal Activity of Essential Oils from *Artemisia negrei*

The antifungal activity of the studied oil was conducted using four fungal species including *Candida albicans* ATCC 10231, *Aspergillus niger* (LBEAH/FS/19), *Aspergillus flavus* (LBEAH/FS/18), and *Fusarium oxysporum* (LBEAH/FS/17). The disk diffusion method was used to achieve this goal as described elsewhere [25]. In brief, Petri dishes with MEA medium were inoculated with *C. albicans*, *A. niger, A. flavus,* and *F. oxysporum*. Next, Whatman paper disks (6 mm in diameter) impregnated with 10 *μ*L of essential oils were placed on the surface of Petri dishes before being incubated at 30°C in the darkness. The inhibition diameter, as well as inhibition percentage, was determined after 48 h of incubation for *C. albicans* and after 7 days for *A. niger, A. flavus,* and *F. oxysporum* [26, 27].

### 2.7. Statistical Analysis

The obtained results were expressed as means ± SEM of triplicate assays. Statistical analysis was conducted using the ANOVA test. A significant difference was statistically considered when *p* < 0.05.

## 3. Results and Discussion

### 3.1. Phytochemical Compounds of Essential Oil

The obtained results showed that the yield of essential oil of *A. negrei* was 1.2%. The highest percentage of essential oil of the genus *Artemisia* was recorded for *Artemisia cana* (1.3%) and *Artemisia frigida* (1.5%). However, the essential oil yield of the aerial part of *Artemisia absinthium*, *Artemisia biennis*, *Artemisia dracunculus,* and *Artemisia ludoviciana* ranges from 0.3% to 0.5%, which is lower than that of *A. negrei* [28]. This yield can be considered important in comparison with some plants that are industrially exploited as a source of essential oils such as rose (0.1–0.35%), rosemary (1–2.5%), peppermint (0.5–1%), neroli (0.5–1%), lavender (0.8–2.8%), aniseed (1–3%), and thyme (2–2.75%) [29].

**Figure 1:**
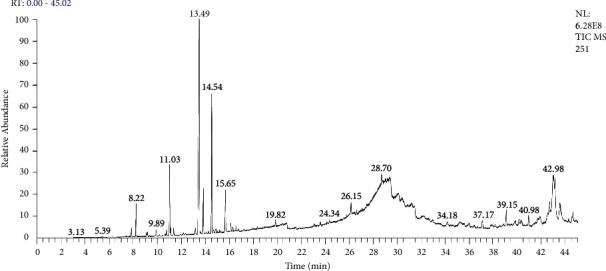
GC-MS chromatographic profile of essential oil from *A*. *negrei*.

The chromatographic analysis of essential oil of *A. negrei* from the folded Middle Atlas revealed the presence of 34 volatile constituting 99.91% of the total essential oil recovered from fresh material (Figure 1; Table 1). The chemical analysis showed that the characterized essential oil possessed many potentially bioactive substances including thujone (29.02%), 2-bornanone (14.68%), octacosane (14.02%) eucalyptol (5.60%), endoborneol (3.78%), bicyclo (3.1.0) hexan-3-on (3.63%), pentacosane (3.07%), and camphene (2.38%). Some compounds identified in the current oil (*β*-thujone, *α*-thujone, borneol, camphor, and 1.8-cineol) were also identified in closer plant species including *Artemisia herba-alba* L., *Artemisia pontica L.*, *Artemisia absinthium L.* [25–27]. Thujone as a major element in the studied oil has been largely identified in essential oils of plants that are used for food and/or medicinal purposes [30].

### 3.2. DPPH Free Radical Scavenging Activity

**Figure 2:**
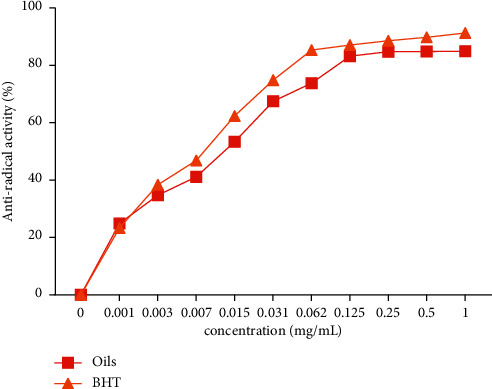
DPPH radical scavenging activity of *A. negrei* essential oil.

The results obtained showed that the studied oil exhibited a potent DPPH free radical scavenging activity (IC50 = 0.0164 ± 0.0011 mg/mL) when compared to BHT (0.0082 ± 0.002 mg/mL) (Figure 2). Our oil with IC_50_ = 0.0164 ± 0.0011 mg/ml is relatively better than that found for oil extracted from *Artemisia dranculus* growing in Turkey, which showed IC_50_ = 100.200, 400, and 1000 *µ*g/ml [31], and *Artemisia herba alba* from southwest Tunisia (IC_50_ = 50.00 *μ*g/mL) [32]. As shown in Table 1, *A. negrei* essential oil contains a higher amount of oxygenated monoterpenes, so that it can be a promising source of radical scavenging agents [33].

### 3.3. Total Antioxidant Capacity

In the current research work, the obtained findings showed that the essential oil from *A. negrei* had promising total antioxidant capacity with a value of 867.71 ± 30.21 mg/g when compared to 472.29 ± 6.19 mg/g of BHT and 307.65 ± 13.08 mg/g of quercetin. It has been indicated that the antioxidant activity of essential oils is closely related to compounds with hydroxyl functions such as alcohols, and phenolic compounds [30, 31].

### 3.4. *ß*-Carotene Discoloration Test

**Figure 3:**
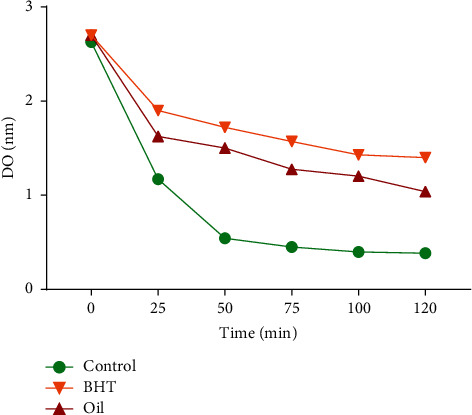
Antioxidant activity of *A. negrei* oil by *ß*-carotene discoloration test.

The evaluation of the antioxidant activity of the extract by using different assays is largely appreciated for comparison purposes [34]. It is thus fitting that the *ß*-carotene bleaching method was used to achieve this goal. A follow-up of the *ß*-carotene oxidation reaction in the presence of the oil and the standard reference (BHT) was performed by measuring the intensity of *ß*-carotene color at a wavelength of 470 nm. As shown in Figure 3, the results reported in percentages indicate that *A. negrei* oil exhibited potent inhibitory capacity (74.1428%) when compared to BHT (100%). The test used showed that *A. negrei* oil was a good hydrogen donor so that it is capable of being a free radical scavenger to control oxidation [35].

The investigated results in this work showed that *A. negrei* exhibited strong antioxidant power that may result from thujone as a major compound detected in the studied oil by GC-MS analysis [36]. In this sense, thujone (*α* and *β*) has been reported to have anti-free radical activity as reported elsewhere [37]. The findings obtained in this work are consistent with those reported elsewhere [38], which demonstrated that essential oil from genus *Artemisia* revealed antioxidant activity of DPPH and ABTS assays. Many works have investigated the relationship between the phytochemical content of the essential oil and the antioxidant potential. In this sense, it was reported that antioxidant power is closely related to the presence of chemicals with hydroxyl function [39]. Therefore, the oil higher in phenolic compounds along with terpene alcohols can have a strong antioxidant effect [40]. According to the results obtained, the essential oils of *Artemisia* species showed a very high antioxidant efficacy even at the lowest concentration tested, so that we can confirm that this potent activity is explained by the richness of the oil in oxygenated monoterpenes.

### 3.5. Antibacterial Activity of Essential Oils of *Artemisia negrei*

**Figure 4:**
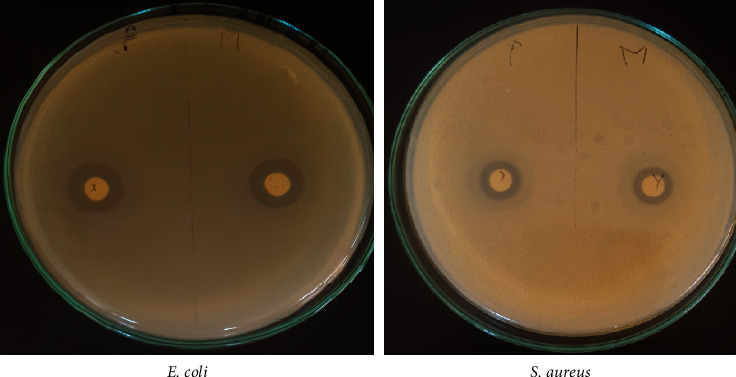
Photograph of Petri dishes showing inhibition zone of essential oils from *A.negeri* against bacterial strains.

Faced with the problems of antimicrobial resistance to synthetic antibiotics, much work has been conducted on the antimicrobial power of natural products including essential oils of certain plants. In this research study, the antimicrobial power of *A. negrei* essential oil was tested vs. five strains including *E. coli 57*, *E. coli 97*, *K. pneumoniae*, *P. aeruginosa,* and *S. aureus* as multidrug-resistant bacteria with a high frequency of contamination and pathogenicity [22]. In this study, the antibacterial activity was evaluated by using inhibition zone diameter (Table 2) and MIC bioassays (Table 3). The results obtained showed that our plant oil had potent antibacterial activity towards *E. coli 57*, *E. coli 97*, *K. pneumoniae*, *P. aeruginosa,* and *S. aureus* since the maximum inhibition zones and MIC values were around 18–37 mm and 1.56–12.5 mg/mL, respectively, towards these species (Figure 4). 1.56 *µ*g/mL was sufficient to inhibit the growth of *P. aeruginosa* (Gram negative), which was the most sensitive to the studied oil followed by *E. coli 9, E. coli 57*, and *K. pneumonia* (Gram negative), which were completely inhibited by 6.25 *µ*g/mL. On the other hand, *S. aureus* (Gram+) was seriously inhibited at a concentration of 12.5 *µ*g/mL. All strains were found to be resistant to the tested antibiotics except for *S. aureus* (Gram+), which was found to be highly sensitive to *Streptomycin* with an inhibition zone diameter of 9.32 ± 0.84 mm and resistant to ampicillin. The antibacterial effect of the oil from *A. negrei* can be explained by the presence of oxygenated monoterpenes identified in oil particularly thujone, eucalyptol, endoborneol, 2-bornanone, and *ß*-terpineol reported to possess interesting pharmacological activities [41]. These findings were proven by Kordali [31], who showed that the oils of closer species such as *Artemisia santonicum* and *Artemisia spicigera* possessing a high level of bioactive oxygenated monoterpenes. On the other hand, a wormwood *A. campestris*, which is mainly composed of hydrocarbon monoterpenes, revealed a weak antimicrobial activity against pathogenic germs such as *E. coli* and *S. aureus* [42]. For getting the antimicrobial effect, antibiotics need to reach and interact with specific target sites. However, the antimicrobial agent is frequently interrupted due to the intervention of different mechanisms in bacteria, which lead to the failure of antimicrobial agents, so that bacteria continue to develop strongly [43]. The low sensitivity of Gram-negative bacteria to antibiotics may result in an outer membrane covering the cell wall, which interacts with the diffusion of hydrophobic agents through the lipopolysaccharide coating. Essential oil from natural sources can successfully cross the cell walls of bacteria and the cytoplasmic membrane inducing disorders of macromolecules (fatty acids, polysaccharides, and phospholipids) [44]. In this work, essential oil from *A. negrei* has almost closer activity vs. Gram-positive as much as Gram-negative bacteria. Hence, we could confirm that essential oil from *A. negrei* is a potent weapon to fight multidrug-resistant strains.

The obtained findings demonstrated that the bacterial strains tested were found to be resistant to antibiotics ampicillin and streptomycin. These results were in accordance with those investigated in previous literature [43], which revealed that *Enterobacter* spp., *S. aureus*, *P. aeruginosa, K. pneumoniae*, *A. baumannii* along with *E. coli* pathogens were too drug-resistant microbes. Moreover, the strains tested in this research work are classified belonging to multidrug-resistant as reported in previous works [38, 40].

### 3.6. Antifungal Activity of Oil from *A. negrei*

Regarding the *in vitro* evaluation of the antifungal activity of *A. negrei* essential oil, the disk diffusion method revealed antifungal activity with a percentage inhibition of 32.93 ± 0.53%, 33.80 ± 1.27%, and 33.66 ± 0.44 against *F. oxysporum*, *A. niger,* and *C. albicans*. However, the essential oils did not show antifungal activity against *A. flavus* when compared to other strains. These results are in agreement with investigated elsewhere [45], which showed that oil from *Borojoa patinoi* Cuatrec exhibited an inhibitory effect towards *C. albicans* since both share some common chemicals. Moreover, numerous studies place priority natural products especially essential oil for controlling fungal strains including *F. oxysporum*, *A. niger*, *A. flavus,* and *C. albicans* [26]. Several epidemiological studies have been conducted on yeast infections indicated that *Candida* is responsible for many diseases [46]. Thus, the use of alternative treatment from a natural source can serve society to control fungal diseases at low cost.

Previously reported literature on the mechanism of action of essential oil towards fungi showed that essential oils are higher in thymol, and p-cymene penetrates cells inducing membrane damage [47, 48]. The reported activities in this work were consistent with the chemical composition of monoterpenes, which are the most potentially responsible for cell membrane damage. In previous works, it was reported that the fungicidal effect of thymol and p-cymene oil on *Candida* spp., resulting in indirect damage to the cytoplasmic membrane of target bacteria [49].

## 4. Conclusion

The present work aimed to shed light on the chemical composition and antioxidant, antibacterial, and antifungal activities of essential oil from *A. negrei* growing in the folded Middle Atlas, Morocco. The results obtained showed that the oil recovered from the studied plant was rich in potentially active compounds. The oil had potent antioxidant, antibacterial, and antifungal activities. Therefore, the oil from *A. negrei* can be used as a valuable natural source for further research that may lead to developing a new weapon to fight free radical damage and microbial resistance.

## Figures and Tables

**Table 1:** Phytochemical components identified in *A. negrei* essential oil by GC-MS.

Peak	RT (min)	Compound Name	RI		Molecular Formula	Area (%)
			Obs	Lit		
1	7.84	*α*-Pinene	938	939	C10H16	0.61
2	8.22	Camphene	965	959	C10H16	2.38
3	9.17	*β*-Pinene	976	979	C10H16	0.29
4	9.89	Myrcene	988	990	C10H16	0.37
5	10.78	Cymene	1024	1024	C10H14	0.44
6	11.03	Cineole	1031	1031	C10H18O	5.60
7	11.11	Limonene	1029	1029	C10H16	0.50
8	11.36	Fenchone	1086	1086	C10H18O	0.50
9	13.16	Artemisia alcohol	1073	1083	C10H18O	0.50
10	13.49	*β*-Thujone	1111	1114	C10H16O	29.02
11	13.82	*α*-Thujone	1100	1102	C10H16O	3.63
12	14.54	Camphenol	1110	1113	C10H16O	14.68
13	15.65	Borneol	1169	1169	C10H18O	3.85
14	16.11	Limonen-4-ol	1662	1667	C10H18O	0.56
15	16.54	*α*-Terpineol	1132	1133	C10H18O	0.35
16	19.85	Bornyl acetate	1286	1288	C12H20O2	0.51
17	20.81	Geranyl formate	1291	1298	C11H18O2	0.88
18	26.15	*α*-Copaene	1376	1376	C15H24	1.00
19	28.70	Aromadendrene epoxide	1641	1641	C15H24O	1.20
20	29.43	Cycloisolongifol-5-ol	1510	1513	C15H26O	2.88
21	30.12	*α*-Acoreno	1630	1633	C15H26O	1.30
22	31.44	*γ*-Muurolene	1476	1479	C15H24	1.17
23	37.17	Curcuphenol	1716	1718	C15H22O	1.09
24	39.15	Hexadecanoic acid	1957	1960	C16H32O2	1.72
25	40.19	Coumarin	1793	1434	C17H28O2	0.65
26	40.99	Trihydroxy benzaIdehyde	1818	1819	C7H6O4	1.05
27	41.77	Isopropyltetradecanoate	1823	1829	C17H34O2	0.56
28	41.91	Isotorquatone	1884	1845	C15H22O4	1.43
29	42.56	Lanceol acetate	1854	1855	C17H2602	0.95
30	42.69	Thujopsenic acid	1863	1864	CI5H2202	1.54
31	43.02	Octacosane	2798	2800	C28H58	14.02
32	43.57	Pentacosane	2497	2500	C25H52	3.07
33	44.56	Octadecanoic acid, ethylester	2122	2125	C20H40O2	0.60
34	44.64	Hexadecanol	1874	1875	CI6H34O	1.01
Total identified						99.91%
Monoterpene hydrocarbons						4.59%
Oxygenated monoterpenes						58.69%
Sesquiterpene hydrocarbons						2.17%
Oxygenated sesquiterpenes						9.44%
Others						25.02%

RT: retention time (min); RI: retention indices; Obs: calculated retention indices of phytochemicals found in *A. negrei* essential oils; Lit: retention indices of phytochemicals found in the literature.

**Table 2:** Diameter of the zone of inhibition in mm by the agar diffusion method.

Compound	Gram-negative bacteria				Gram-positive bacteria
	*E. coli 57*	*E. coli 97*	*K. pneumoniae*	*P. aeruginosa*	*S. aureus*
EO	37.21 ± 1.24	28.37 ± 3.21	19.05 ± 2.01	18.51 ± 0.91	23.41 ± 2.36
Streptomycin	—	—	—	—	9.32 ± 0.84
Ampicillin	—	—	—	—	—

**Table 3:** Minimum inhibitory concentration (MIC in mg/mL).

Compound	Gram-negative bacteria				Gram-positive bacteria
	*E. coli 57*	*E. coli 97*	*K. pneumoniae*	*P. aeruginosa*	*S. aureus*
EO	6.25 ± 0.7	6.25 ± 0.7	6.25 ± 0.61	1.56 ± 0.20	12.5 ± 1.03
Streptomycin	4.51 ± 0.04	5.27 ± 0.23	3.38 ± 0.01	—	6.21 ± 0.04
Ampicillin	—	—	—	—	—

## Data Availability

Data used to support the findings are included within the article.
